# Techniques for Studying Decoding of Single Cell Dynamics

**DOI:** 10.3389/fimmu.2019.00755

**Published:** 2019-04-11

**Authors:** Stevan Jeknić, Takamasa Kudo, Markus W. Covert

**Affiliations:** ^1^Department of Bioengineering, Stanford University, Stanford, CA, United States; ^2^Allen Discovery Center for Systems Modeling of Infection, Stanford, CA, United States; ^3^Department of Chemical and Systems Biology, Stanford University, Stanford, CA, United States

**Keywords:** dynamics, decoding, encoding, single cell, signaling, live cell microscopy

## Abstract

Cells must be able to interpret signals they encounter and reliably generate an appropriate response. It has long been known that the dynamics of transcription factor and kinase activation can play a crucial role in selecting an individual cell's response. The study of cellular dynamics has expanded dramatically in the last few years, with dynamics being discovered in novel pathways, new insights being revealed about the importance of dynamics, and technological improvements increasing the throughput and capabilities of single cell measurements. In this review, we highlight the important developments in this field, with a focus on the methods used to make new discoveries. We also include a discussion on improvements in methods for engineering and measuring single cell dynamics and responses. Finally, we will briefly highlight some of the many challenges and avenues of research that are still open.

## Introduction

Specificity requires a cell to be able to recognize heterogeneous signals as inputs and reliably compute heterogenous outputs in response. Cells receive signals that can be derived from the organism itself—autocrine, paracrine, and endocrine signals—from the environment, or from other organisms, for example, during infection. Frequently, several signals are present simultaneously and in rapidly changing amounts and durations. Despite this, cells must be able to reliably differentiate signals and generate a specific response based on the signal identity, intensity (i.e., the amount of signal present), frequency (i.e., the duration the signal is present for), and context (i.e., the other signals present and the cellular state).

Accordingly, cells have evolved myriad mechanisms to receive, transmit, and process information reproducibly in a fluctuating and noisy environment. Cells generally first “encode” the signal they receive, by transmitting information about the signal into the activation of specific signaling pathways. Cells are then able to “decode” this information into phenotypic responses and changes in gene and protein expression. Notably, stochastic fluctuations in the concentration of signaling molecules, the numbers of intracellular signaling proteins, and the composition of the microenvironment can be substantial at the single cell level (1). Therefore, the pathways have evolved to be robust to this unavoidable biological noise. The signaling pathways are also frequently redundant or overlapping; many cellular signaling pathways are able to transmit information from a variety of signals to produce heterogeneous outcomes, while many signals can affect multiple pathways (2–4).

**Figure 1:**
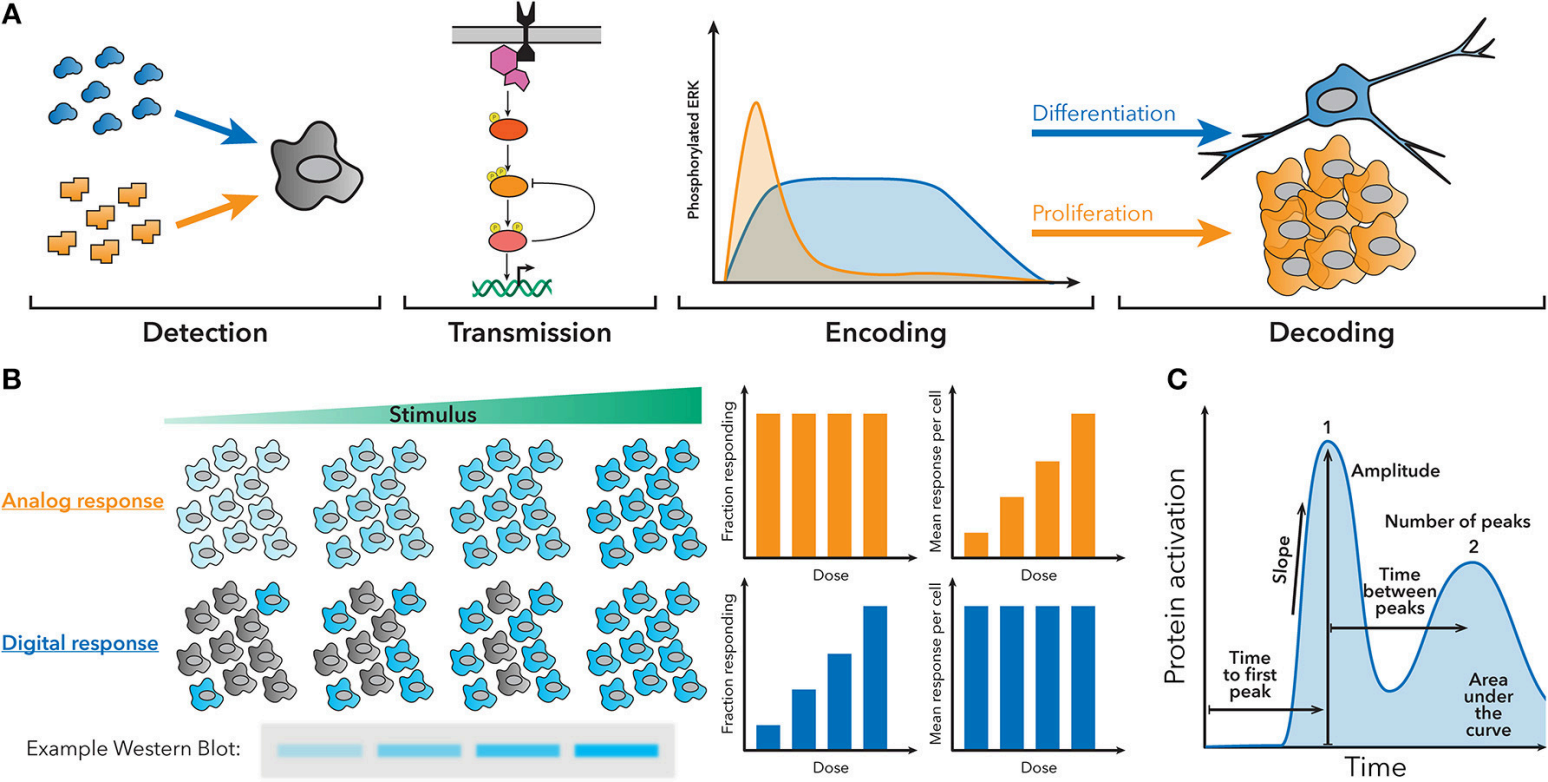
Fundamentals of dynamic encoding and decoding. **(A)** Cells can encode information about the signals they encounter as dynamic patterns of signaling pathway activation. These patterns can then be decoded to produce a specific response. For example, NGF creates sustained ERK activation, which leads to differentiation, while EGF creates transient ERK activation, which leads to proliferation. **(B)** Population-level measurements, such as a western blot, can hide the behavior of single cells in the underlying system. For example, an analog or digital response could produce similar western blots, despite having different amounts of active cells and activity per cell. **(C)** Examples of some features of dynamic traces that can be used to encode information.

Over the past couple decades, it has become increasingly clear that cells use a variety of signaling architectures to encode detailed information about the signals they encounter as temporal patterns of activation of transcription factors, kinases, calcium ions, and other signaling molecules (5–7). Cells must therefore also possess mechanisms to interpret this dynamic information and translate it into a transcriptional or phenotypic response. One classic example is the discrimination of nerve growth factor (NGF) and epidermal growth factor (EGF) by rat neuronal precursors. NGF stimulation produces sustained activation of the ERK pathway that prompts the cell to differentiate, while EGF stimulation engenders transient activation of the ERK pathway that is decoded as a proliferative cue (Figure 1A) (8–10). Another classic example is the nuclear factor (NF)-κB innate immune signaling pathway, which exhibits oscillatory activation patterns when stimulated with tumor necrosis factor (TNF)-α, but sustained activation when stimulated with lipopolysaccharide (LPS), resulting in different gene expression patterns (11–13).

The importance of dynamic decoding of stimulus dose and identity has also been described in several other pathways. For example, p53 is known to use dynamics to differentiate doses of gamma radiation, and between gamma and UV radiation (14, 15). The Msn2 pathway in yeast uses dynamics to differentiate between osmotic and oxidative stress, as well as the severity of glucose starvation (16). Moreover, the Notch pathway has been recently discovered to differentiate between some Delta-like ligands using dynamic patterns (17). Finally, recent studies have described how individual cells interpret multiple, simultaneous stimulants (18, 19). These are only a few examples in a large space; we strongly encourage readers to reference some of the excellent reviews published on cellular encoding and decoding for a more complete overview, especially of classic examples (20–22).

Clearly, dynamic encoding and decoding are widespread in biology, but studying how cells interpret cellular dynamics can be challenging, because population measurements occlude the behavior of individual cells (Figure 1B), and making targeted perturbations in signaling pathways is difficult. In order to understand how individual cells encode and decode dynamics, often multiple different measurements have to be made in the same single cell at multiple timepoints. Features of the signaling dynamic patterns (Figure 1C) can then be correlated to other measurements of cell behavior to understand how the cell is using dynamics. Recently, the portfolio of high-throughput and single-cell technologies has expanded greatly and become accessible to a wider spectrum of labs, allowing signaling dynamics to be studied in myriad systems.

For example, optogenetics has enabled precisely targeted activation of signaling pathways allowing for greater understanding of the role of dynamics in development and cancer signaling (23–26). Advances in microfluidics have enabled new precision in stimulation timing and dosage (19, 27–29), as well as studies of single cell protein secretion or transcriptomics (30–33). Finally, new reporters have created opportunities to make measurements in novel signaling pathways and contexts (29, 34–41). In this review, we will highlight various experimental strategies that have been successfully used to study cellular dynamic decoding, with an emphasis on single cell studies. We will also discuss recent technological developments that have enabled the field to grow rapidly, and end by discussing some potential future avenues of study and technological challenges that still persist.

## Dynamic Decoding of Cellular Information

Population-level studies have revealed some of the connections between signaling dynamics and cellular responses (12, 13, 42, 43). However, as discussed previously, population measurements are not necessarily indicative of single cell behavior (Figure 1B). In order to understand how single cells decode dynamic signals into a phenotypic response, it is necessary to make combined measurements of the signaling dynamics and the downstream cellular response in the same single cell. Microscopy has proven to be an invaluable tool for these studies, given the versatility of measurements that it can make, including not only live-cell fluorescence for measuring the signaling dynamics themselves, but also single molecule fluorescence *in situ* hybridization (smFISH) for measuring gene expression (44), and immunofluorescence or other antibody-based methods for measuring protein expression. In addition, there has been recent work to combine other modalities, such as RNA-seq and microfluidics, with live-cell imaging, expanding the repertoire of possible measurements. Here we present a collection of recent studies that demonstrate effective strategies for probing the connection between dynamics and cellular responses on a single cell level.

### Live-Cell Imaging Coupled With Measurements of Physical Phenotypes

The most straightforward way to interrogate how cells decode dynamics is to measure signaling dynamics and clear phenotypic responses, such as cell death, cell migration, or cell division. These measurements are well-adapted to live-cell microscopy, as measurements of cellular dynamics and the phenotypic response can be made using the same measurement modality with few technical limitations.

**Figure 2:**
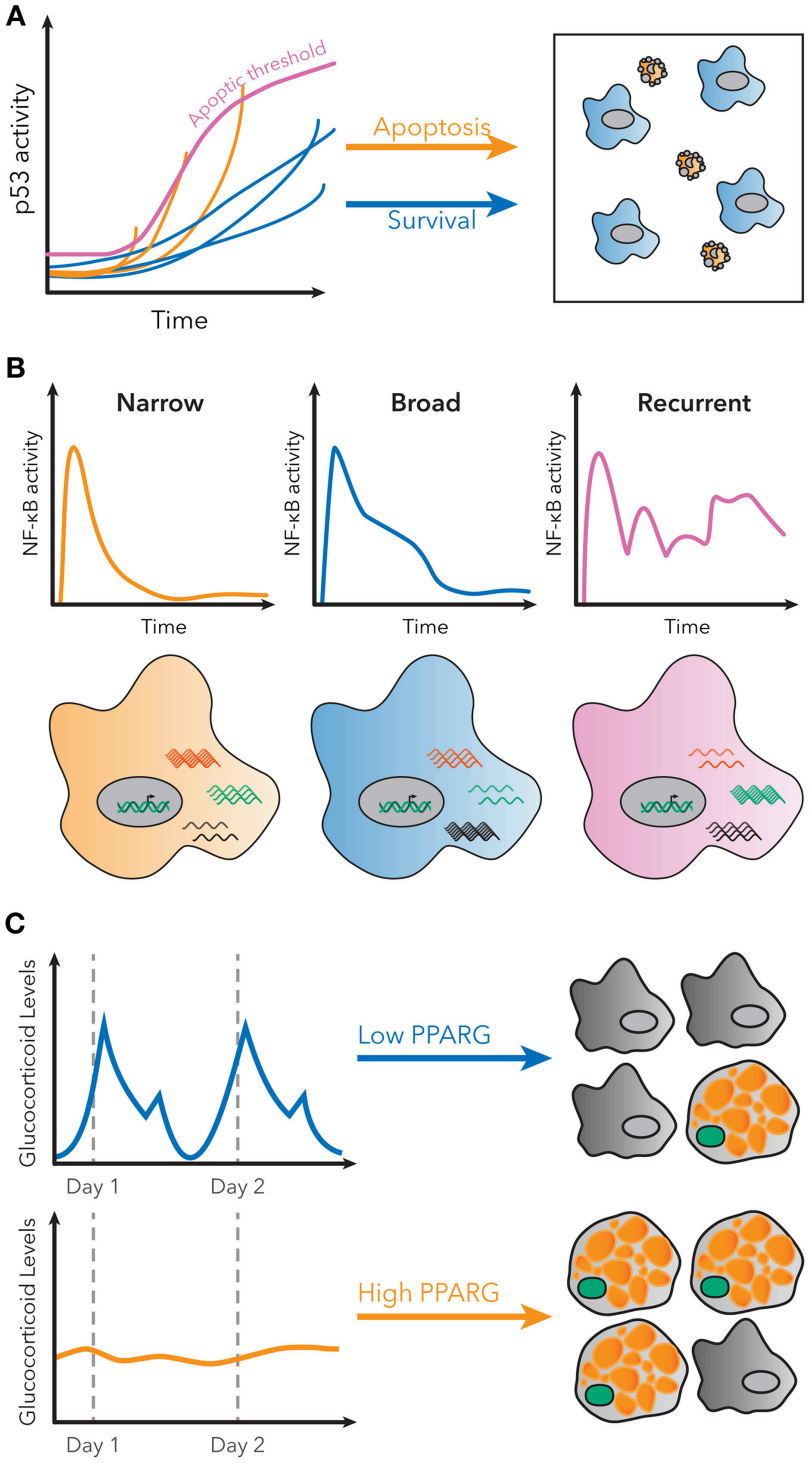
Examples of decoding dynamic signaling patterns. **(A)** Apoptosis due to p53 signaling is not determined by a static threshold, but by a dynamic, increasing threshold. Some cells do not undergo apoptosis, even though they have higher p53 levels than some cells that do undergo apoptosis. Figure adapted from Paek et al. (47). **(B)** Subpopulations with distinct patterns of NF-κB activity exist in single cells stimulated with LPS. These patterns are correlated with different gene expression patterns for known NF-κB targets. Figure adapted from Lane et al. (32). **(C)** Basal rates of adipocyte differentiation are low *in vivo*, despite large pulses of glucocorticoid production daily. However, continuous glucocorticoid inputs of similar total magnitude induce more stabilization of PPARG, indicated in green, and higher differentiation rates. Figure adapted from Bahrami-Nejad et al. (73).

For example, p53 is a transcription factor with a critical role in regulating cell growth and apoptosis in response to DNA damage (45). Previous population-level studies suggested cells with p53 activation below a specific threshold would initiate growth arrest, while cells above that threshold would undergo apoptosis (46). However, single cell studies using a fluorescent p53 reporter showed that in order to undergo apoptosis, p53 levels in the cell must indeed reach a threshold, but that this threshold increases over time (Figure 2A) (47). Therefore, the decision of apoptosis or cell growth arrest is determined by the dynamics of p53 activation, as opposed to a static threshold. This observation could only have been made using a single cell dynamical approach.

A similar study revealed aspects of TNF signaling that are correlated with apoptosis. TNF signaling initiates a pro-apoptotic cascade, as well as induction of pro-survival genes by NF-κB (48). Using a microfluidic device to precisely control stimulus timing and dosage, Lee et al. showed that short pulses—as short as 1 min—of TNF-α can be more effective at inducing apoptosis than longer pulses. Single-cell measurements of NF-κB activation showed that longer pulses of TNF sustained longer residence times of NF-κB in the nucleus, suggesting that NF-κB dynamics are correlated with the relative balance of pro-apoptotic and pro-survival signaling (49). The pro-apoptotic arm of the pathway is initiated on a slower time-scale than, and is inhibited by, the pro-survival arm of the pathway (48, 50, 51). Therefore, the authors propose a model where the sustained NF-κB activation caused by longer TNF pulses, maintains inhibition of the pro-apoptotic signaling arm, leading to greater relative pro-survival signaling (49).

Live-cell microscopy has also been used to understand how the spatiotemporal dynamics of the mitogen-activated protein kinases (MAPKs) regulate mating processes in single yeast cells. In order to undergo successful mating and fusion, individual yeast cells must remodel their cell walls, arrest the cell cycle, and polarize their growth (52, 53). A Förster resonance energy transfer (FRET) reporter of the activity of two MAPKs, Fus3 and Kss1, coupled with visual observation of cell shape and growth, revealed that elevated Fus3 activity at the sites of polarized growth were required for initiating polarity and fusion between mating cells, showing that the spatiotemporal patterning of Fus3 activity, and not just Fus3 levels, are required for the correct mating phenotype (54).

Reporters of multiple different pathways can also reveal how the context of an immune stimulus can affect how an immune cell will respond (55). Innate immune cells use pattern recognition receptors (PRRs), such as Toll-like receptors (TLRs), to detect molecules that are indicative of an infection. A cell line expressing both an NF-κB and a c-Jun N-terminal kinase (JNK) reporter was challenged with increasing levels of immune stimulation, from only LPS to infection with *Salmonella typhimirium*, allowing for measurements of the cell's signaling response in each case (35). This work showed how an individual cell uses TLR signaling to discriminate similar signals in a variety of different contexts. For example, in a population of cells exposed to *S*. *typhimirium*, uninfected cells typically activated only NF-κB, while those cells that were infected with bacteria typically activated both JNK and NF-κB (35).

Multiple live-cell reporters exist that broaden the types of physical phenotypes that can be directly measured using live-cell microscopy. For example, fluorescent probes have been developed that allow for quantitative measurements of single cell kinase activation (34, 37), visualization of various modes of cell death and caspase activation (36, 56), as well as cell cycle progression and measurements of proliferation rate (57). This proliferation reporter was used in conjunction with a FRET-based reporter of ERK activity to show that cells use ERK pulse duration and overall activity to regulate entry into S-phase and cell cycle timing. Subsequent experiments using high content immunofluorescence (HCIF) suggested that the main quantitative factor controlling steady-state proliferation in single cells is ERK output (58).

These experiments are clear examples of ways that we can begin to understand how signaling dynamics generate physical phenotypic responses. Furthermore, the development of new biosensors, improvements in our ability to regulate cellular dynamics (see “Experimental strategies for engineering or modulating dynamic signaling patterns”), and improvements in high-throughput phenotype characterization (59), should allow for even more insight in this area of research.

### Live-Cell Imaging Coupled With Measurements of Gene Expression and Transcription

Signaling pathways often exert control over the cell by making changes to the expression of specific target genes. In such cases, dynamic signaling patterns are likely to be decoded as quantitative changes in gene expression. However, unraveling this connection can be more challenging than measuring physical phenotypes because it requires measuring signaling dynamics and gene expression in the same single cell. Notwithstanding this complication, many recent studies have demonstrated experimental strategies to successfully combine measurement modalities to uncover interesting results.

For example, the nuclear abundance of NF-κB varies significantly in both stimulated and unstimulated cells, suggesting that NF-κB transcription might also be highly variable (60). However, smFISH measurements in single cells show that transcript levels for many NF-κB targets vary less than the activity of NF-κB (61). Measurements of a fluorescent NF-κB reporter, combined with end-point smFISH quantification of NF-κB regulated transcripts, showed that dynamic quantities, such as fold change of NF-κB, were better predictors of transcriptional levels than static quantities, such as NF-κB nuclear abundance. Subsequent mathematical modeling revealed a potential mechanism based on an incoherent feed-forward loop generated by transcription factor competition (61). Recent work, also using a smFISH-based strategy, further showed that fold change of NF-κB was an accurate predictor of transcript levels at promoters with both high and low levels of TNF-induced transcription (62).

A similar example involved studying the responses of NF-κB to simultaneous LPS and TNF-α stimulation to understand how cells respond to multiple stimuli. A fluorescent reporter was used to measure NF-κB activation in 3T3 cells across a range of LPS and TNF-α concentrations. For most concentrations, the response could be classified as an LPS-only or TNF-α-only response, but for a number of intermediate concentrations, there was a synergistic response that had characteristics of both TNF- and LPS-stimulated cells. Subsequent smFISH measuring the mRNA of a number of relevant chemokines and cytokines revealed that dual-responding cells had, on average, higher expression of Cxcl10 and Csf3 (18). Similar strategies have also been successfully used in other signaling systems (63–65).

Single-cell dynamic imaging can also reveal new phenomena that can be further studied using other techniques. For example, the HIV virus is known to integrate into the host genome and lay dormant in some T lymphocytes, which presents a major obstacle for treatment with antiretroviral therapy (66). Although these latent reservoirs of virus exhibit stochastic, low level activation, the molecular regulators controlling viral activation are still incompletely understood (67). NF-κB is a known regulator of the HIV long terminal repeat (LTR) promoter; however, a recent study using fluorescent reporters of HIV and NF-κB activation revealed that NF-κB activation is not predictive of levels of viral activation across different clones (68). Instead, using smFISH and chromatin immunoprecipitation (ChIP), the authors found that the chromatin environment regulates transcriptional bursting and can explain clone-to-clone variability. These findings revealed that NF-κB-chromatin interactions are required to explain transcriptional bursting and viral activation (68).

FISH-based strategies have also been used to elucidate the role dynamics can play *in vivo* during development. For example, it had been shown that myogenesis requires transient, not sustained, activation of Notch, but the mechanism of transient Notch activation was not clear (69). Recently, it was revealed that the Notch pathway also uses dynamics to encode and decode information about the identity of the activating stimulus (17). A creative experimental system, using engineered “sender” cell lines that produce either Dll1 or Dll4 and a “receiver” cell line with a chimeric Notch receptor driving expression of a fluorescent protein allowed measurements of the dynamics of Notch activation in the presence of either ligand. These experiments revealed that Dll1 stimulation leads to pulsatile Notch activation, while Dll4 creates sustained Notch activation, with differences in gene expression as a result. The results were reproduced in an *in vivo* model by electroporating either Dll1 or Dll4 into one side of the neural crest of a chick embryo and then using hybridization chain reaction (HCR) FISH to stain for MyoD1, a muscle regulatory factor. Their results revealed that MyoD1 is upregulated by Dll1, which creates pulsatile dynamics, while Dll4, which creates sustained dynamics, downregulated MyoD1 (17).

In systems where endpoint measurements of gene expression are insufficient, multiple fluorescent reporters can be used to measure signaling and transcriptional output simultaneously. For instance, TNF-α is a known regulatory target of NF-κB, that can subsequently regulate downstream responses through paracrine and autocrine signaling (11, 32, 70). A cell line with reporters for both NF-κB activity and transcription from the TNF-α promoter was used to simultaneously measure the signaling and transcriptional dynamics in real-time. Measurements revealed low correlation between many measures of NF-κB activity and the output from the TNF-α promoter. However, the time-integrated NF-κB activity was well correlated with the total output from the TNF-α promoter, demonstrating that continuous measurements of transcriptional activity can reveal more information than endpoint measurements in some systems (71).

Finally, it is now also possible to measure signaling dynamics and genome-wide transcriptional responses in the same single cell. RNA-seq provided a method to measure the entire transcriptome of a single cell, but it remained unsolved how to connect those data with measurements of transcription factor activation dynamics. Lane et al. used microfluidics to isolate cells for live-cell imaging and single-cell RNA-seq to connect the identity of the cell in both datasets. Their results revealed that distinct patterns of NF-κB signaling in response to the same stimulus correlated to different global transcriptional responses (Figure 2B) (32). The ability to measure global gene expression resultant from heterogeneous dynamics is exceedingly useful, because it allows for phenotypic characterization of single cell dynamics without a need for *a priori* knowledge of the target genes.

### Live-Cell Imaging Coupled With Protein Expression Measurements

Frequently, a cellular response to signals that it receives is to differentially regulate the expression or secretion of proteins. For example, a large part of the immune response is coordination of cytokine and chemokine secretion by immune cells at the site of infection. Therefore, another promising avenue for research in cellular dynamics is to study changes in protein expression and secretion in conjunction with measurements of dynamics. Immunofluorescence can be used to measure intracellular protein expression, similar to measurements of gene expression using smFISH. Alternatively, microfluidic devices or microwell-based assays can be used to measure protein secretion from single cells.

For example, protein quantification can be used to understand how signaling pathways in cells control differentiation. Hormones such as glucocorticoids strongly induce adipogenesis *in vivo* and *in vitro*, but basal rates of preadipocyte differentiation are low in living animals, despite large daily spikes in glucocorticoid hormone production (72). This raises the question of how the differentiation pathway in preadipocytes is able to filter daily, pulsatile signals. Live cell imaging of endogenous adipogenic transcription factors CEBPB and PPARG, and staining for markers of fat cell differentiation, revealed that the transcriptional circuit in preadipocytes effectively filters out pulsatile signals, but responds to continuous signals of the same total magnitude (Figure 2C). A model predicted that such a response could be achieved if the system had both fast and slow feedback loops, and further protein and mRNA staining revealed FABP4 as a potential slow-feedback partner in the pathway (73).

Most often, protein expression is measured at the experiment's endpoint, but sometimes more frequent measurements of downstream protein expression changes are required. ERK signaling has been described as both a “persistence detector,” which drives approximately digital expression of target genes based on the duration of ERK activity (74–76), and also as a system where peak amplitude qualitatively regulates gene induction (77). Simultaneously measuring ERK activity and induction of Fra-1, a target of ERK, using live-cell reporters instead revealed that linear integration of ERK activity was the primary determinant of downstream responses (78).

Single-cell protein secretion is more challenging to measure, because of the low amounts of protein secreted and the need for isolating individual cells. One strategy for studying single cell protein secretion is to use total internal reflection microscopy to measure secreted proteins in a microwell by a sandwich immunoassay (79). This approach was used to make concurrent measurements of caspase-1 activation using a FRET reporter and IL-1β secretion in single cells. Further analysis revealed that caspase-1 activation is digital and controls a burst of IL-1β secreted from dead macrophages (33). Alternatively, multiple microfluidic strategies to combine live-cell imaging and antibody-based detection of secreted proteins have been developed (30, 31). Cells are initially captured in single cell wells, where they can be exposed to precise doses and durations of stimuli and imaged using a fluorescent microscope. The media from each cell's well can be sampled and measured with antibodies for secreted proteins at various time points during the experiment. Depending on the device, it is also possible to stain cells using immunofluorescence, allowing for both secreted and intracellular proteins to be measured (30).

Finally, changes in protein expression can also be controlled by chromatin regulators, which impart histone and DNA modifications (80, 81). It is now possible to study how single cells use different chromatin regulators to produce varying dynamics of gene expression. Bintu et al. used a doxycycline-inducible system to recruit individual chromatin regulators to regulate the expression of a fluorescent protein. They showed that epigenetic silencing and reactivation are digital processes in single cells and that different chromatin regulators modulate the fraction of cells silenced. Further, using a stochastic model, they describe the different dynamics for both silencing and reactivation, created by each chromatin regulator (82). How cells use chromatin modifications to process signal information is still poorly understood (83), but studies of single cell chromatin regulation dynamics provide a promising avenue for future research.

## Experimental Strategies for Engineering or Modulating Dynamic Signaling Patterns

Studying cellular decoding is more challenging than studying cellular encoding, for technical and biological reasons. The primary biological challenge is that cells have complicated signaling pathways that interact with each other and control heterogeneous outputs. Thus, it is technically difficult to prove that the dynamics are the causative factor in the phenotypic measurement. It has also been difficult to perturb signaling dynamics in ways that would help to establish causality of phenotype, especially in single cells. Here we summarize strategies that have been successfully used to modulate signaling dynamics, as well as significant technical advances that have enabled novel ways of controlling signaling dynamics in single cells.

### Engineered Dynamics Using Optogenetics and Other Synthetic Systems

**Figure 3:**
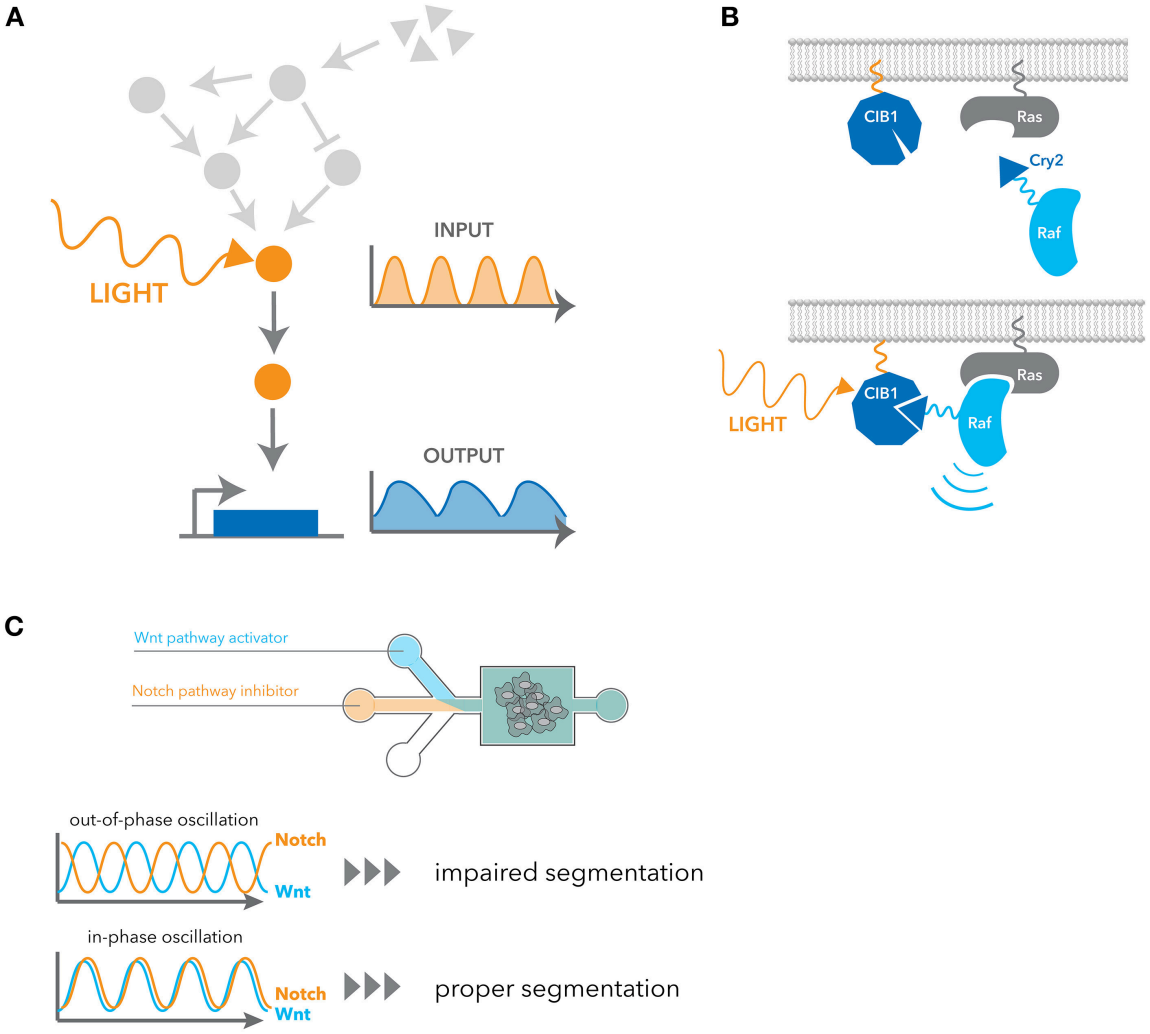
Engineering approaches for manipulating dynamic signaling patterns. **(A)** Optogenetic tools can dynamically and selectively activate a pathway in isolation from endogenous receptor signaling contexts. **(B)** Blue light induces dimerization between the N-terminal CIB1 and Cry2 domain fused to cRaf, leading to the recruitment of cRaf to the membrane. Ras activates cRaf at the membrane, and thus it activates the downstream ERK pathway. **(C)** Microfluidic devices were used to control the flux of small-molecule inhibitors of the Notch and Wnt signaling pathway. In-phase oscillations of these two pathways led to proper mesoderm segmentation, whereas out-of-phase oscillation impaired segmentation.

Recent advancements in synthetic biology have opened exciting new ways of precisely and selectively controlling dynamic signaling. One such advancement is the field of optogenetics, which exploits light to control protein function and cell activities with high spatio-temporal resolution (Figure 3A). Optogenetic tools are generally faster and more selective than pharmacological stimulation, and their ability to generate flexible temporal patterns brought us a concept of engineering system identification to study the characteristics of cellular signaling pathway in a more direct manner. Here we introduce a few examples of optogenetic strategies applicable to signaling dynamics, but more detailed information about limitations and other applications have also been recently reviewed (84–86).

One commonly used system is Phy-PIF, an optogenetic system with a fast deactivation rate. Both binding and dissociation are induced by light stimulation; red light induces binding and infrared light induces dissociation (87). OptoSOS adopts this system to activate the Ras-ERK signaling pathway by inducing translocation of SOS to the plasma membrane. It can be used to reproducibly activate the pathway using very short pulses (< 1 min) and high frequencies of activation, enabling one to measure the frequency response of the pathway (88). This system was recently used to show that a mutation in B-Raf in a human cancer line led to slower decay kinetics of the pathway, meaning that a larger space of input frequencies and strengths are interpreted as growth signals in this cell line (23).

Cry2 is another widely used optogenetic system. It has a slower activation and deactivation rate compared to other systems, but it has the unique property that it exhibits both hetero- and homo-dimerization upon blue light stimulation. Cry2 binds to the N-terminal domain of CIB1 in a blue-light dependent manner. Similarly to OptoSOS, cRaf fusion to Cry2 was used to activate the ERK pathway by inducing translocation to the plasma membrane (89) (Figure 3B). Using this system, it was shown that pulsatile ERK activation led to higher cell proliferation than sustained activation. Several genes that are induced better by pulsatile ERK activation were also identified. Photoactivation by recruiting a partner to a membrane comprises many examples such as PKA (90), AKT (26, 91) and TrkA (92). In addition to this heterodimerization between Cry2 and CIB1, Cry2 is known to have a propensity for oligomerization. This property of oligomerization was later improved with a small change in sequences (93, 94). As many signaling events are initiated by homo-oligomerization, particularly receptor signaling, they have been widely applied to many signaling pathways (95–100).

A light-oxygen-voltage-sensing (LOV) domain is a photosensory motif found in many proteins across diverse species. Blue light stimulation induces covalent bond formation between the LOV domain and its flavin cofactor, leading to a partial unfolding between the LOV domain and C-terminal *A*-helix. LOV domains have been engineered for many applications due to the small size of this domain (~110 amino acids). For example, a light-switchable gene promoter system was developed by fusing a fungal LOV domain and the Gal4 transcription factor lacking the dimerization domain (101). This system was also applied to control temporal patterns of proneural gene Ascl1 expression in neural progenitor cells (24), and the oscillatory and sustained expression of Ascl1 were shown to induce proliferation and differentiation, respectively.

Contrary to the examples above exploiting translocation or recruitment, there are many optogenetic tools that can directly control allostery and fragment complementation. This includes Dronpa-based strategies (25, 102), LOV domain-based proteins utilizing photo-uncaging (103–106), and a number of light-sensitive channels and receptors. For example, Hannanta-Anan and Chow used melanopsin to generate a wave of calcium release (107). By systematically controlling the calcium oscillation amplitude, frequency, and duty cycles, they found downstream NFAT integrates total elevated calcium concentrations due to its slow export rate.

Dimerization can also be induced chemically; the dimerization of FKBP and FRB with rapamycin is a classic example that has been used in a wide variety of applications for many years (108–110). Though many tools are essentially irreversible due to high affinity binding, acute induction of dimerization or translocation has been an effective strategy to investigate causation of signaling events. For instance, Santos et al. constructed the nuclear Cdk1-FKBP and cyclin B1-FRB reporters to test the spatial positive feedback regulation of Cdk1-cyclin B1 (111). The chemical dimerization of these complexes in nuclei triggered cyclin B1 nuclear translocation, which was confirmed by translocation of a fluorescent protein fused to cyclin B1, but not fused to FRB.

Another potential approach to control dynamics is to engineer a fully synthetic version of the pathway in an orthogonal cellular environment. As an example, the ERK pathway was reconstructed in yeast, and this minimal cascade itself was shown to generate ultrasensitivity (112). Recently, a synthetic NF-κB signaling pathway was introduced in yeast to study its oscillatory behavior (113). The amplitude and period of the oscillatory response to α-factor can be experimentally regulated by tuning the level of RelA from an inducible promoter, the stability of the protein, and/or the promoter strength driving the expression of the negative feedback component, IκBα. Synthetic systems provide an easy way to manipulate pathway parameters and circuit structures with small-molecule inputs.

### Microfluidics Can Precisely Control Dose and Timing of Stimulus

Fluidic control was a standard approach to dynamically manipulate a stimulation pattern before genetic or synthetic approaches became popular. A simple fluidic setup with a pump has been used for several decades to control input flux, including early studies of glucagon signaling (114) and calcium signaling (115). Microfluidic devices now represent a dramatic improvement, providing us with more precise control to generate virtually any kind of temporal pattern, to study both dynamic encoding and decoding.

NF-κB activation in single cells has been well studied using such devices (116, 117). One study showed that cells respond to TNF-α in a probabilistic manner, meaning that only a fraction of cells activates the NF-κB pathway when TNF-α concentrations are low. Microfluidics-based temporal control of TNF stimulation enabled multiple discrete pulses of TNF-α to be delivered to the media. These experiments showed that variability in the NF-κB response depends not only on pre-existing cellular variability, but also on a previously unknown stochastic element (118).

In terms of dynamic decoding, the filter characteristics of the yeast stress pathway has been extensively studied using microfluidic devices (16, 119, 120). By modulating the amplitude, frequency, and duration of periodic Msn2 nuclear translocation, it was shown that each promoter transcribed by Msn2 has a distinct sensitivity to amplitude and pulse frequency. These differences can differentially regulate at least four classes of genes downstream of Msn2 (121).

Microfluidics have also been used to manipulate cellular phenotypes by controlling signaling dynamics. For example, EGF and NGF stimulation in PC12 cells activate the ERK pathway in a transient and sustained manner, respectively, leading to different cellular outcomes. With a microfluidic device, Ryu et al. inverted the outcomes from each growth factors simply by changing the stimulus patterns (28). As another example, Sonnen et al. observed that the segmentation of the presomitic mesoderm is dependent on relative timing between Wnt and Notch signaling oscillations (29). They used microfluidics to generate either in-phase or out-of-phase oscillations of these two pathways and showed the out-of-phase oscillations impair segmentation (Figure 3C).

## Technical Improvements in High Throughput Single Cell Measurements

Many of the examples above show the versatility and capability of microscopy to interrogate relationships between signaling dynamics and downstream phenotypes in single cells. It is becoming clear that signaling dynamics can be decoded to distinct gene expression programs leading to diverse cellular phenotypes; yet most studies were only able to measure a few genes due to technical challenges. Here we will go over some of the recent technical advancements that potentially expand the throughput and accessibility of this measurement modality.

As we saw in the examples above, FISH and immunofluorescence are commonly used techniques for capturing downstream responses. FISH can be implemented in a high-throughput manner, as one can strip or bleach probes and thus iterate detection (122–124). The downside of these techniques is their cost and sensitivity, since many probes are required to bind one species of mRNA and thereby amplify a specific signal. Two recently developed methods utilize different signal amplification schemes, enabling higher sensitivity and gain. The first method is proximity ligation *in situ* hybridization technology (PLISH) (125). PLISH amplifies a target region by rolling circle amplification after generating closed circle probe oligonucleotides by RNA-templated proximity ligation. The second technique, called click-amplifying FISH (clampFISH), uses non-enzymatic click chemistry to generate closed circle oligonucleotides (126, 127). Both techniques were shown to provide better fluorescence signals in both cell culture and tissue samples (126).

Similar to high-throughput FISH approaches, there are methods which attempt to determine the amount of many specific proteins via immunofluorescence over multiple cycles. For example, Lin et al. developed cycIF by bleaching a fluorophore conjugated with a primary antibody with hydrogen peroxide (128). They were able to detect 60 proteins in a tumor tissue sample by repeating the bleaching and staining steps for each protein of interest (129). Another group developed the antibody elution method called 4i which elutes antibodies with a mix of reducing agent, low pH and chaotropic salts, and a blocking buffer (130). This is compatible with indirect immunofluorescence, and 40 proteins were detected with this approach. A similar approach, co-detection by indexing (CODEX), uses oligonucleotide conjugated antibodies (131). Instead of repeating antibody binding steps, this method first carries out the binding process, followed by repeated detection of the barcoded antibodies by incorporating fluorophore-labeled nucleotides by polymerase.

These methods rely on fluorescence detection and thereby the number of detections at a time is limited by the spectral overlap. In contrast to these approaches, imaging mass cytometry and multiplexed ion beam imaging use metal-labeled antibodies (132–134). The signal from the metal isotopes are measured via mass spectrometry, allowing simultaneous detection of more proteins than would be possible by fluorescence. Both methods were able to measure more than 30 proteins from tumor samples (135, 136), and it can be also combined with high-throughput FISH methods (137).

For live-cell image analysis, computational automation is increasingly a requirement due to the amount of data that can readily be acquired. One of the recent breakthroughs in this area involves deep learning. Convolutional neural networks were first applied to classification of histopathologic images for a diagnostic purpose (138–140). In 2016, a software tool called DeepCell employed this type of classification task for automated image segmentation of cells (141). In addition to a higher segmentation accuracy for fluorescent images, this deep learning-based approach also allows us to segment objects using a non-labeled image such as phase-contrast or DIC images (142–144).

## Concluding Remarks

The capability and compatibility of single-cell measurement techniques has advanced significantly in the last several years. It is now possible to measure signaling pathway activation and RNA, protein or metabolite levels in single cells, often in real time. The throughput of these measurements is also increasing rapidly, especially with the development of better computational techniques for image analysis and iterative FISH and immunofluorescence approaches. Moreover, our ability to engineer single cell dynamics is also rapidly improving with the development of techniques such as optogenetics. As a result, studies describing how individual cells respond to inputs using dynamic patterns have expanded into new systems and levels of detail.

Practically, these new discoveries may reveal ways to target signaling dynamics in disease contexts, potentially leading to novel treatments (145). Pharmacologically altered signaling dynamics have only been demonstrated in a few studies, but this may change with the development of better tools and knowledge (64, 146). Additionally, better understanding of dynamic cellular responses can help lead the way to cells with functional engineered signaling circuits, which have large potential as possible therapeutics and scientific tools (147).

Nonetheless, this field is still in the early stages and many challenges remain to be addressed. Reporter development remains a challenging problem, and thus reporters currently exist for only a small subset of pathways. Furthermore, most measurements of signaling pathways continue to rely on exogenous reporters, which can differ from the responses seen with endogenous proteins. While work has been done to make it easier to directly measure endogenous proteins, these methods still remain more difficult than using exogenous reporters. Finally, large-scale genetic screens have enabled new levels of understanding in many fields through the ability to search for important effectors across the entire genome. However, using microscopy in conjunction with genome-wide screens is still exceedingly challenging because of the need to connect the measurements made using microscopy to the genetic perturbation in each cell. Thus, there is still much room for improvement in both the techniques available to the study of dynamics, as well as the number of systems that these techniques can be applied to.

## Author Contributions

SJ, TK, and MC designed and wrote the manuscript.

### Conflict of Interest Statement

The authors declare that the research was conducted in the absence of any commercial or financial relationships that could be construed as a potential conflict of interest.
